# Validation of Minimally-Invasive Sample Collection Methods for Measurement of Telomere Length

**DOI:** 10.3389/fnagi.2017.00397

**Published:** 2017-12-06

**Authors:** Stephanie A. Stout, Jue Lin, Natalie Hernandez, Elysia P. Davis, Elizabeth Blackburn, Judith E. Carroll, Laura M. Glynn

**Affiliations:** ^1^Department of Psychology, University of Denver, Denver, CO, United States; ^2^Department of Biochemistry and Biophysics, School of Medicine, University of California, San Francisco, San Francisco, CA, United States; ^3^Department of Psychiatry and Human Behavior, University of California, Irvine, Irvine, CA, United States; ^4^Cousins Center for Psychoneuroimmunology, University of California, Los Angeles, Los Angeles, CA, United States; ^5^Department of Psychology, Chapman University, Orange, CA, United States

**Keywords:** telomere length, dried blood spot, saliva, venous blood, cellular aging, concordance, qPCR

## Abstract

**Objective**: The discovery of telomere length (TL) as a biomarker of cellular aging and correlate of age-related disease has generated a new field of research in the biology of healthy aging. Although the most common method of sample collection for TL is venous blood draw, less-invasive DNA collection methods are becoming more widely used. However, how TL relates across tissues derived from these sample collection methods is poorly understood. The current study is the first to characterize the associations in TL across three sample collection methods: venous whole blood, finger prick dried blood spot and saliva.

**Methods**: TL was measured in 24 healthy young adults using three modes of sample collection for each participant: venous whole blood, finger prick dried blood spot and saliva. Relative TL was measured using quantitative polymerase chain reaction.

**Results**: TL in finger prick dried blood spots (DBS) washighly correlated with TL in whole blood (*r* = 0.84, *p* < 0.001). Salivary TL was also correlated with whole blood TL (*r* = 0.56, *p* = 0.005), but this association was not as strong as that of dried blood spot TL (Steiger’s *Z* = 2.12, *p* = 0.034). TL was longer in saliva than in whole blood or DBS (*p*’s < 0.001).

**Conclusions**: These findings have important implications for future study design by supporting the validity of less-invasive methods that can be implemented with vulnerable populations or in the field. Further, these findings aid in interpreting the burgeoning area of biological aging research and may shed light on our understanding of inconsistencies in the empirical literature.

## Introduction

Telomeres are repetitive DNA-protein structures that cap the ends of chromosomes and protect DNA from degradation and damage (Armanios and Blackburn, 2012). Telomeres shorten with each cell division, contributing to a net loss of telomere length (TL) across the lifespan (Armanios and Blackburn, 2012). As a hallmark indicator of biological aging (López-Otín et al., 2013; Kennedy et al., 2014), TL has provided researchers with critical insights into the origins of disease risk and longevity. Specifically, shorter TL in leukocytes predicts the incidence and severity of cardiovascular disease, cancer and mortality risk as well as longevity (Cawthon et al., 2003; Epel et al., 2008; Willeit et al., 2011; Haycock et al., 2014; Rode et al., 2015). Given that shortening of TL likely plays an important role in disease etiology (Campisi and d’Adda di Fagagna, 2007; Armanios and Blackburn, 2012), it has become a widely investigated biomarker in research concerning the biology of health. A variety of different DNA sample collection methods are used across studies, limiting the ability to make comparisons of telomere dynamics across studies.

In humans, TL has been most extensively studied in leukocytes or peripheral blood mononuclear cells (PBMCs) from venous whole blood samples (see Epel et al., 2008; Willeit et al., 2011; Blackburn et al., 2015). In addition to the ease of obtaining large quantities of high-quality DNA, whole blood collection for leukocyte or PBMC TL is often the preferred method because they are linked to poor health outcomes, and both reflect the replicative potential of the immune system as well as effects of systemic factors on telomere maintenance in other tissues (Blackburn et al., 2015). However, collecting whole blood samples is labor intensive, expensive, and this invasive procedure may not be feasible with sensitive populations (e.g., infants, children) or in research settings where blood cannot be processed immediately (e.g., in the field). For this reason, less invasive DNA collection methods such as collection of dried blood spots (DBS) and saliva samples have been implemented to measure TL (Mitchell et al., 2014; Drury et al., 2015; Lapham et al., 2015). Despite the expanding use of less invasive tissue collection techniques, the relation between TL measured across these sample collection methods is poorly understood and the validity of measuring TL in DBS or saliva is not well-established.

To interpret and reconcile the expanding body of literature that explores the clinical and biological significance of TL, it is of increasing importance to characterize the relations among different methods of DNA collection for TL measurement. Few studies have explored whether TL in tissues collected using less invasive methods is related to venous blood TL. Mitchell et al. (2014) examined the associations between TL in saliva and whole blood leukocytes among 16 adult volunteers. They found that saliva TL and leukocyte TL were strongly positively correlated (*r* = 0.72). Zanet et al. (2013) conducted a study characterizing how different forms of collection, processing and storage of specimen impacted TL. They report that TL of venous blood stored as whole blood was highly correlated to the TL of that same venous blood sample stored as DBS (*r* = 0.86). These findings demonstrate that TL is minimally influenced by the DBS storage method. However, they do not address the validity of collecting DBS by finger prick for TL measurement. To our knowledge, there are no studies that compare TL across these three collection methods: venous whole blood, finger prick DBS and saliva.

The current study addresses gaps in the literature by measuring TL in three tissue samples collected from a healthy, non-clinical population. We will characterize the magnitude of the correlation and differences in relative TL across saliva, finger prick DBS and venous whole blood samples.

## Materials and Methods

### Participants

Participants included 24 healthy undergraduates (12 females) from 18 to 20 years of age (*M* = 18.4) recruited from a small University in Southern California. Participants were primarily non-Hispanic white (*n* = 15) and included Latino/a (*n* = 6), Asian (*n* = 2) and multi-ethnic (*n* = 1) individuals. This study was carried out in accordance with the recommendations of the Institutional Review Board of Chapman University with written informed consent from all subjects. All subjects gave written informed consent in accordance with the Declaration of Helsinki. The protocol was approved by the Institutional Review Board of Chapman University. Based on a recent study (Mitchell et al., 2014), which found that saliva TL and leukocyte TL were correlated at *r* = 0.72, we determined that with a sample size of 24, we have greater than 95% power to obtain a similar effect size when *α* < 0.05.

### Procedures

Participants completed a single laboratory visit in which a phlebotomist collected venous whole blood, finger prick DBS and saliva samples. Although buccal swabs are a common non-invasive collection method, they were not assessed in the current study because saliva samples yield higher quality DNA than a buccal swab (Hansen et al., 2007; Rogers et al., 2007).

#### Venous Whole Blood DNA Collection

Whole blood was collected through venous draw into heparinized tubes (5 ml). After inverting 30–40 times, 1 ml was pipetted into five 1 ml screw cap tubes and immediately stored at −80°C. Genomic DNA was purified from 200 μl of whole blood using QIAamp DNA Blood Mini Kit (QIAGEN, Hilden, Germany).

#### Dried Whole Blood Spot DNA Collection

Five blood spots were collected directly from fingertip prick onto Whatman 903 Protein Saver Cards. The cards were left to dry for a minimum of 4 h before being placed in a sealable Whatman foil bag with desiccant and stored at −20°C. Genomic DNA was purified from dried whole blood spots from six 3 mm diameter punches using QIAamp DNA Investigator Kit (Cat#56504, QIAGEN, Hilden, Germany). The average yield from spot samples was 59.1 ng (SD = 15.8 ng).

#### Saliva DNA Collection

A 2 mL sample of liquid saliva was obtained by participants spitting into the Oragene saliva DNA collection kit (tj Genotek Inc. Ottawa, ON, Canada) containing 1 mL of DNA stabilizing liquid. The samples were stored at room temperature until processing for DNA extraction. Genomic DNA was purified from 500 μl saliva using Agencourt DNAdvance Genomic DNA Isolation kit (Beckman Coulter). The average yield from saliva was 6.03 μg (SD = 1.42 μg). Although buccal swabs are a common, less invasive DNA collection method for TL measurement, only saliva TL was examined in the current study because saliva collection has a higher DNA yield and the quality of DNA obtained is superior to that of buccal swab (Hansen et al., 2007; Rogers et al., 2007).

### Telomere Length Measurement by qPCR

Relative TL values for all biological samples were determined using the monoplex quantitative polymerase chain reaction (qPCR) method established by Cawthon (2002). This method has been validated against the Southern blot method, which measures absolute TL, as a precise assay for determining TL within the same subject samples, with consistency across laboratories (Aviv et al., 2011), and in large cohort studies (Lapham et al., 2015). This method measures the ratio of the telomere repeat copy number to the single-copy gene copy number (T/S ratio) in the study sample as compared to reference DNA samples. The primers for the telomere PCR are *tel1b* [5′-CGGTTT(GTTTGG)_5_GTT-3′], used at a final concentration of 100 nM and *tel2b* [5′-GGCTTG(CCTTAC)_5_CCT-3′], used at a final concentration of 900 nM. The primers for the single-copy gene (human beta-globin) PCR are *hbg1* [5′ GCTTCTGACACAACTGTGTTCACTAGC-3′], used at a final concentration of 300 nM, and *hbg2* [5′-CACCAACTTCATCCACGTTCACC-3′], used at a final concentration of 700 nM. The final reaction mix contains 20 mM Tris-HCl, pH 8.4; 50 mM KCl; 200 M each dNTP; 1% DMSO; 0.4× Syber Green I; 22 ng *E.coli* DNA; 0.4 Units of Platinum Taq DNA polymerase (Invitrogen Inc.) per 11 microliter reaction; 1–10 ng of genomic DNA. Tubes containing 26, 8.75, 2.9, 0.97, 0.324 and 0.108 ng of a reference DNA (from Hela cancer cells) were included in each PCR run so that the quantity of targeted templates in each research sample can be determined relative to the reference DNA sample by the standard curve method. The same reference DNA was used for all PCR runs. The telomere thermal cycling profile consists of: Cycling for T (telomic) PCR: denature at 96°C for 1 min, 1 cycle; denature at 96°C for 1 s, anneal/extend at 54°C for 60 s, with fluorescence data collection, 30 cycles. Cycling for S (single-copy-gene) PCR: denature at 96°C for 1 min, 1 cycle; denature at 95°C for 15 s, anneal at 58°C for 1 s, extend at 72°C for 20 s, eight cycles; followed by denature at 96°C for 1 s, anneal at 58°C for 1 s, extend at 72°C for 20 s, hold at 83°C for 5 s with data collection, 35 cycles.

To control for inter-assay variability, eight control DNA samples were included in each run. In each batch, the T/S ratio of each control DNA was divided by the average T/S for the same DNA from 10 runs to get a normalizing factor. This was done for all eight samples and the average normalizing factor for all eight samples was used to correct the participant DNA samples to get the final T/S ratio. The T/S ratio for each sample was measured twice. When the duplicate T/S value and the initial value varied by more than 7%, the sample was run the third time and the two closest values were reported. The average CV is 2.1% for whole blood DNA, 2.7% for saliva DNA and 6.6% for dried blood spot DNA.

### Statistical Analyses

We conducted Pearson correlations to assess the concordance of T/S ratios across venous blood, saliva and DBS. We used two-tailed Steiger’s *z* tests (Steiger, 1980; Lee and Preacher, 2013) to compare the strength of these associations across collection methods. Differences in the T/S ratio between collection methods were determined with a series of paired *t-tests*.

## Results

**Figure 1:**
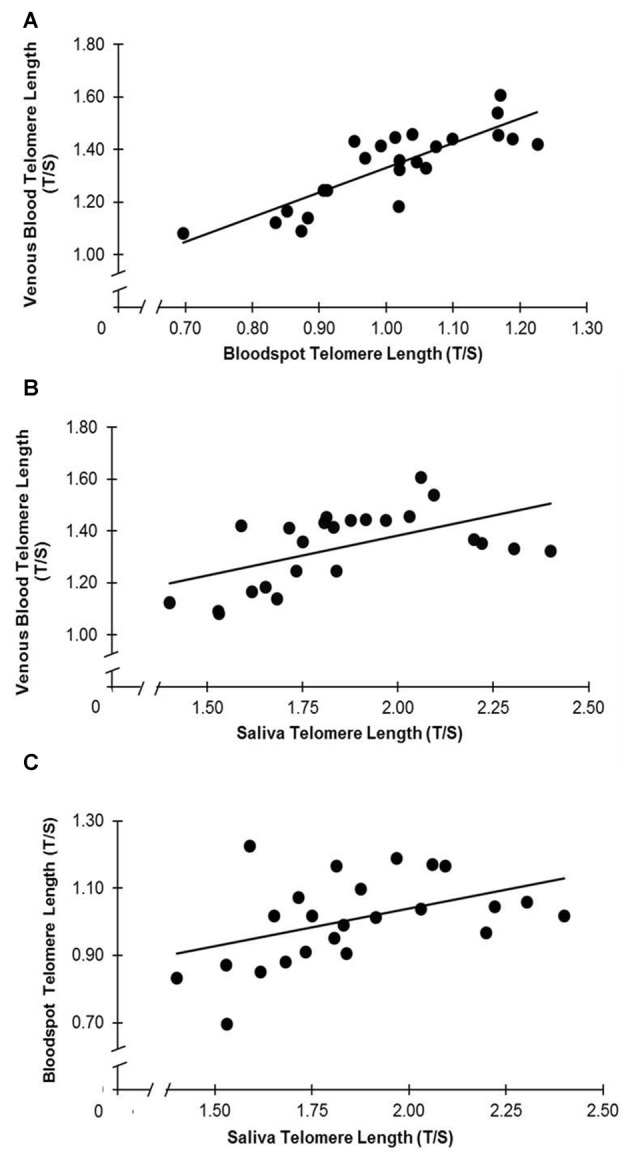
**(A–C)** Telomere length (TL) is associated across collection types. **(A)** T/S in venous blood and dried blood spots (DBS) are correlated (*r* = 0.84, *p* < 0.001). **(B)** T/S in saliva and venous blood are correlated (*r* = 0.56, *p* = 0.005). **(C)** T/S in saliva and DBS are correlated (*r* = 0.45, *p* = 0.027).

The associations between relative TL (T/S ratio) in venous blood, dried whole blood spot and saliva can be seen in Figure 1. DBS T/S and venous whole blood T/S were highly correlated (*r* = 0.84, *p* < 0.001; *R*^2^ = 0.70; 95% CI: 0.65, 0.93). Salivary T/S and venous whole blood T/S were also positively associated (*r* = 0.56, *p* = 0.005; *R*^2^ = 0.31; 95% CI: 0.20, 0.78). In addition, there was a positive association between salivary T/S and DBS T/S (*r* = 0.45, *p* = 0.027; *R*^2^ = 0.20; 95% CI: 0.05, 0.72). Steiger’s *Z* analyses revealed that the correlation between venous whole blood and DBS T/S was stronger than the correlation between venous blood and salivary T/S (Steiger’s *z* = 2.12, *p* = 0.034), suggesting that finger prick DBS TL provides a closer approximation of whole blood TL than does salivary TL.

**Figure 2:**
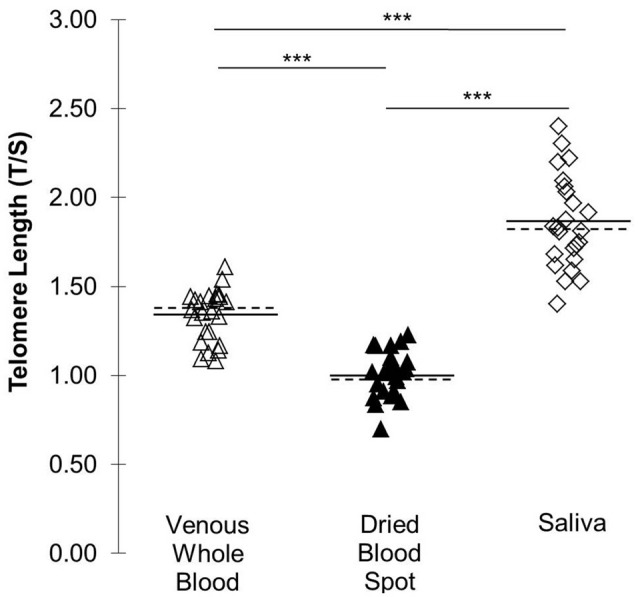
TL (T/S) across three DNA collection methods. Solid lines indicate *mean* T/S by collection type. Dotted lines indicate *median* by collection type. ***p < 0.001.

Figure 2 displays differences in TL across sampling methods. The T/S ratio was higher in saliva than in venous whole blood (*t*_(23)_ = 11.72, *p* < 0.001; 95% CI: 0.43, 0.61) and DBS samples (*t*_(23)_ = 17.86, *p* < 0.001; 95% CI: 0.75, 0.95). The venous whole blood T/S ratio was significantly higher than DBS T/S (*t*_(23)_ = −20.16, *p* < 0.001; 95% CI: −0.36, −0.30).

## Discussion

This study provides critical evidence regarding the concordance of relative TL (i.e., T/S) across venous whole blood, finger prick DBS and saliva in a healthy non-clinical sample. Our findings suggest that relative TL in venous whole blood is better approximated by DBS TL than by saliva TL. However, relative TL was associated across all three sample collections, demonstrating that T/S measurement in DBS and saliva are appropriate, less-invasive alternatives to venous whole blood collection.

We found that DBS TL was more closely associated with venous whole blood TL than was TL in saliva. In contrast to the study by Zanet et al. (2013), which tested the effect of different storage methods on TL in the same venous blood sample, the current study provides a direct comparison between finger prick DBS TL and venous whole blood TL. The fact that our correlation of *r* = 0.84 between finger prick DBS TL and venous whole blood TL is comparable to the *r* = 0.86 correlation between venous DBS TL and whole blood TL described previously (Zanet et al., 2013) suggests two things. First, finger prick DBS TL is a good approximation of venous whole blood TL. Second, the variation between these sample collection methods is largely attributable to differences in storage method rather than collection site (venous vs. finger capillary). We also showed that TL in saliva is positively associated with TL in venous whole blood, which has been reported previously (Mitchell et al., 2014). The associations across the three sample collection methods are not surprising given the cell populations within each sample type; TL measured in both finger prick blood and venous blood is primarily derived from leukocytes, and saliva contains both leukocytes and epithelial cells at varying proportions (Aps et al., 2002; Theall et al., 2013).

We additionally evaluated whether the T/S ratio differed across the three collection methods. We observed the longest TL in saliva, consistent with prior work showing saliva TL to be longer than that of whole blood (Mitchell et al., 2014). We also observed shorter TL in finger prick DBS than in venous whole blood, which is contrary to previous work showing DBS TL to be longer than in saliva (Zanet et al., 2013). Because there are several methodological differences between these two studies, further studies would be required to elucidate this discrepancy. Although differences in TL are expected given that the ratio of leukocyte subtypes can differ between finger capillary and venous blood samples (Yang et al., 2001), it is also plausible that the observed differences in T/S across collection types may be attributable to non-uniform residual impurities which can differentially impact the qPCR reactions for the telomere and single-copy-gene.

The current study indicates that finger prick DBS and saliva can both be implemented effectively to measure TL, and are therefore viable alternatives to invasive venous blood draws. Our findings solidify the case for using finger prick DBS in research specifically interested in leukocyte TL. DBS collection from finger prick is minimally invasive, inexpensive, and robust to long-term storage (Mas et al., 2007), and previous work demonstrates that it is possible to successfully extract sufficient, high-quality DNA from DBS for the purpose of TL measurement (Drury et al., 2015). Further, by characterizing relations in TL across these sample collection methods, the current study provides information that is critical to interpreting the existing literature.

We collected specimens from a relatively homogenous sample of healthy young adults, which limits our ability to assess how clinical conditions or other individual differences might affect associations in relative TL across tissue types. For example, it is possible that these relations in relative TL vary as a function of disease risk (Blackburn et al., 2015), age (Frenck et al., 1998), sex (Gardner et al., 2014) and race/ethnicity (Diez Roux et al., 2009). However, this methodological decision also could be viewed as a strength; we detected robust positive associations in TL across the three tissue sample types despite this limited variability, suggesting it is likely that greater variability would increase the magnitude of these associations. Further, the current study conducted in a young, healthy sample can be used to contextualize future work with larger and more heterogeneous samples.

Our study validates the widespread use of less invasive peripheral measures of TL and supports finger prick DBS and saliva collection for TL measurement. The current study also addresses a crucial gap in the literature surrounding concordance across sample collection methods, shedding light on a burgeoning area of research that is elucidating the biological mechanisms of aging.

## Author Contributions

All authors contributed to writing. JL and NH contributed to data collection and processing. SAS, NH and LMG contributed to data analysis. JL, EPD, EB, JEC, LMG contributed to study design.

## Conflict of Interest Statement

JL is a co-founder of Telomere Diagnostics and serves on its scientific advisory board. The other authors declare that the research was conducted in the absence of any commercial or financial relationships that could be construed as a potential conflict of interest.
